# A new vaccination approach for Salmonellosis employing a multi-epitope vaccine based on live microbial cell factory from *Lactococcus lactis*

**DOI:** 10.1016/j.psj.2025.104789

**Published:** 2025-01-07

**Authors:** Reyhaneh Sadat Moosavi-Kohnehsari, Mahnaz Jafari-Sohi, Tohid Piri-Gharaghie, Shakiba Tolou-Shikhzadeh-Yazdi, Mona Aghassizadeh-Sherbaf, Romina Hosseinzadeh

**Affiliations:** a Department of Biology, Faculty of Basic Sciences, East Tehran Branch, Islamic Azad University, Tehran, Iran; b Department of Microbiology, Razi Vaccine and Serum Research Institute, Agricultural Research, Education and Extension Organization (AREEO), Karaj, Iran; c Biotechnology Research Center, Faculty of Biological Sciences, East Tehran Branch, Islamic Azad University, Tehran, Iran; d Department of Biology, Faculty of Sciences, Islamic Azad University, Mashhad Branch, Mashhad, Iran; e Department of Biology, Faculty of Basic Sciences, Islamic Azad University, East-Tehran Branch, Tehran, Iran; f Department of Microbiology, Faculty of Basic Sciences, East Tehran Branch, Islamic Azad University, Tehran, Iran

**Keywords:** Salmonella vaccine, Reverse vaccinology, Live bacterial vectors

## Abstract

A major health and financial burden in the chicken sector is *salmonella* infection. It is difficult to create an oral vaccination that can provide strong intestinal mucosal immunity in birds, particularly cross-protection against several *Salmonella* serotypes. As a result, the poultry industry needs a powerful oral vaccination platform that uses live bacterial vectors to prevent various *Salmonella* serotypes. The genetically engineered L*. lactis* was given orally to birds as a vaccine after a multi-epitope vector was created using a reverse vaccinology technique. After the plasmid was digested, the target group produced a 72 kDa protein called multi-epitop. Birds that received the L*. lactis*/pNZ8121-Multi epitope vaccination showed increased levels of interferon (IFN-γ) and NFkB1α, increased transcription rates of cytokines, and a significant presence of IgY antibodies specific to the multi epitope gene in their serum. *Salmonella* infection is a severe health and economic burden in the poultry industry, according to spleen sections from the L*. lactis*/pNZ8121-Multi epitope. Developing an oral vaccine that can provide birds robust intestinal mucosal immunity—specifically, cross-protection against many *Salmonella* serotypes—is challenging. The results provide a fresh method for creating new immunological candidate multi-epitome genes by using the food-grade, non-pathogenic *Lactococcus lactis* as a protein cell factory. This method provides a unique technique to assess the long-term sustainability, cost, safety, and usefulness of experimental pharmaceutical products.

## Introduction

*Salmonella* is a Gram-negative bacterium, extensively distributed throughout the environment and often located in the gastrointestinal system of livestock and humans (Jajere et al., 2019). According to the standard Kauffmann–White schema for the antigenic categorization of *salmonellae*, there are around 2600 variants that vary in their antigenic composition. *Salmonella* is a pathogenic bacterium that may impact the gastrointestinal system of chickens (Foley et al., 2013). According to the Centers for Disease Control and Prevention (CDC), *Salmonella enterica* accounts for around 1.35 million infections each year, leading to 26,500 hospitalizations and 420 fatalities. Worldwide, Salmonella infections in poultry cause significant economic losses, due not only to reduced productivity but also to the continuous efforts and control measures required to protect public health (Galán-Relaño et al., 2023). Poultry is a common reservoir for Salmonella, presenting a public health risk via its dissemination in the food supply. As a result, international markets impose stringent sanitary requirements and many regulations pertaining to food quality and public health (Hobbs et al., 2010). In the United States and Europe, despite continuous public health and regulatory attempts to prevent and manage Salmonellosis, its incidence has persisted significantly. In EU nations, Salmonellosis ranks as the second most often reported foodborne gastrointestinal illness in people (Schirone et al., 2021). In the United States, non-typhoidal *Salmonella* spp. Accounts for 11 % of all foodborne infections, resulting in 35 % of hospitalizations and 28 % of total fatalities (Williams et al., 2017). *S. Enteritidis* is mostly linked to egg-related salmonellosis outbreaks, whereas *S. typhimurium* is regarded as the primary etiological agent of foodborne Salmonellosis (Moffatt et al., 2016).

Despite the existence of over 2,500 *Salmonella* serotypes in the natural world, just 20 serotypes account for over 82 % of human infections, mostly attributed to *Salmonella Enteritidis, Typhimurium, Newport*, and *Heidelberg* (Tauxe, et al., 1999). In humans, the ingestion of infected chicken meat and eggs accounts for nearly 70 % of salmonellosis cases. Consequently, immunizing poultry versus *Salmonella* is a feasible method and is widely used (Desin, 2013). Vaccination is crucial in biosecurity protocols on poultry farms to avert *Salmonella* infections, enhancing avian resistance to infection and thus diminishing horizontal delivery, fecal excretion, and the incidence of egg contamination. Regrettably, the *Salmonella* vaccines accessible for broilers are few and provide insufficient protection till slaughter (Acevedo-Villanueva et al., 2021). Killed and live attenuated vaccines have been utilized to control *Salmonella* in chicken production, demonstrating broad effectiveness (Hofacre et al., 2021). Primarily, these vaccinations are commercialized; nevertheless, in some nations, accessibility may fluctuate according to local sanitary registration procedures (Weil-Olivier et al., 2014). Killed *Salmonella* vaccines significantly decrease the prevalence of *S. Enteritidis* when administered to laying hen flocks. These vaccinations correlate with a decrease in *Salmonella* levels in feces, internal organs, and eggs, along with reduced mortality, lesions, and clinical manifestations in many animal models (El-Shall et al., 2019). Although inactivated vaccines fail to provoke a protective cell-mediated immune reaction adequately and may lose some bacterial antigens throughout inactivation, they are considered highly safe and do not pose a risk of introducing live vaccine strains into the food supply. The live *Salmonella* vaccinations are deemed dangerous since they may disseminate in the environment and affect the human food supply (Mkangara, 2023).

Conversely, inactivated vaccines elicit suboptimal immunity and protection. Owing to these constraints, less than 1 % of the chicken sector is using a live *Salmonella* spray vaccine. F.D.A. rules explicitly restrict the use of the live *Salmonella* vaccination within 21 days prior to slaughter (Dolatyabi et al., 2024; Abozeid et al., 2023).

An essential strategy for developing secure and effective immunizations versus Salmonellosis involves using live genetically modified vectors, which are benign microorganisms that produce antigens for *Salmonella Enteritidis* and *Salmonella Typhimurium* (Guo et al., 2020). The antigenic combination of *Salmonella Enteritidis* and *Salmonella Typhimurium* can create a potential multi-epitope candidate against these bacteria. Lactic acid bacteria are now used as vectors for the expression of pathogenic antigens in live organisms (Cano-Garrido et al., 2015). Experimental data indicates that the studied bacterial vectors may infect animals and generate pathogenic antigens within the infected cells (Kazemi-Roudsari et al., 2024). *Lactococcus lactis* is a well-known gram-positive bacterium that is often used in the production of probiotic products. It is also the first living genetically modified organism that was used to treat human disease (Wu et al., 2023). Dr. Piri-Gharaghie's research group had previously utilized *Lactococcus lactis* to augment vaccination and successfully induce an immune system against Brucella outer membrane proteins (OMPs) (Piri-Gharaghie et al., 2024).

Despite notable advancements in the development of recombinant vaccines, their application is impeded by limitations like instability, stringent storage conditions, and elevated production and purifying costs (Ghattas et al., 2021). Therefore, the implementation of innovative systems is essential. Multi-epitope vaccines, developed using reverse vaccinology, provide an innovative immunization approach that lacks the disadvantages above (Alizadeh et al., 2022). Multi-epitope vaccines are free from biological contamination since they are produced by chemical synthesis. The multi-epitope vaccines demonstrate aqueous solubility and maintain stability under extreme circumstances. Multi-epitope vaccines are synthesized chemically, guaranteeing their absence of naturally occurring contamination. These vaccines are hydrophilic and can maintain their stability even under adverse settings (Samad et al., 2022). The peptides may be specifically designed for selectivity. Reverse vaccinology involves the replication and expression of all proteins predicted, by computational approaches, to be present on the surface or secreted by an organism's genetic sequence (Zhang et al., 2002). After that, each protein is subjected to high-throughput vaccination to evaluate its ability to elicit antibodies in animal that can efficiently eradicate or neutralize the target pathogen (Rawal et al., 2021).

This study examines the viability of using L*. lactis* for immunizing hens towards *Salmonella Enteritidis* and *Salmonella Typhimurium*. We utilized the recombinant L*. lactis*-pNZ8121 to construct a multi-epitope gene for *Salmonella Enteritidis* and *Salmonella Typhimurium*. The multi-epitope antigen was subsequently delivered orally to hens utilizing L*. lactis*-pNZ8121 and recombinant L*. lactis*-pNZ8121-multi-epitope. Subsequently, we analyzed the immune response. Consequently, we sought to develop a mucosal vaccination using the lactic acid bacterium L*. lactis* as a live microbial carrier for antigen delivery. This procedure proved a beneficial alternative and a safe vaccination strategy against illnesses caused by *Salmonella Enteritidis* and *Salmonella Typhimurium*.

## Materials and methods

### Immunoinformatic section

#### Retrieval of protein sequences

The online proteomics application Vaxign (https://violinet.org/vaxign/) was utilized to examine the proteome of the *Salmonella Enteritidis* and *Salmonella Typhimurium* strains. The complete proteome of these specific bacteria was obtained from the National Centre for Biotechnology Information (NCBI) webpage (https://www.ncbi.nlm.nih.gov/). An extract of the FASTA file was generated employing the chosen protein sequences. The VaxiJen v2.0 website (http://www.ddgpharmfac.net/vaxijen/) was then utilized to assess the immunogenicity of the proteins using the conventional threshold level. The auto-covariance (ACC) conversion method of this service produces prediction results with an accuracy of 70–89 %. Subsequently, the Algpred service (http://crdd.osdd.net/raghava/algpred/) was employed to ascertain the allergenic profiles of the chosen antigens using Blast study on allergen representative peptides (ARPs) section. This webpage uses a unique alignment-free descriptive adjective fingerprint approach, achieving an accuracy of 88.9 %. The Vaxign platform was employed to detect the transmembrane (TM) helix. Protein molecules with a reduced number of TMs have been selected for the subsequent phase of inquiry due to their immunological compatibility and non-allergenic properties.

#### Detection of T Lymphocyte epitopes

The NetCTL v1.2 website (http://www.cbs.dtu.dk/services/NetCTL/) was used to analyze the chosen protein sequences and predict CTL epitopes with elevated combination scores. The threshold level of 0.90 was used. NetMHCIIpan v. 3.2 (http://www.cbs.dtu.dk/services/NetMHCIIpan/) was utilized to forecast helper T cell epitopes among a number HLA-DRB1 alleles, namely, DRB1×01:01, DRB1×03:01, DRB1×07:01, DRB1×09:01, DRB1×10:01, DRB1×11:01, and DRB1×15:01.

Subsequently, the VaxiJen v2.0 website (http://www.ddg-pharmfac.net/vaxijen/), the AllerTOP v2.0 website (https://www.ddg-pharmfac.net/AllerTOP/), and the ToxinPred website (http://crdd.osdd.net/raghava/toxinpred/) were used to evaluate antigenicity, allergenic configuration, and toxicological effects, respectively.

#### Detection of B-lymphocyte epitopes

The LBL epitopes within the chosen data were identified utilizing the ABCpred website (https://webs.iiitd.edu.in/raghava/abcpred/). A 0.80 limit of threshold was selected and epitopes that scored above 0.90 were selected for further analysis. The VaxiJen v2.0, AllerTOP v2.0 and ToxinPred v2.0 were utilized to assess the antigenic, allergic, and toxic characteristics of the anticipated LBL epitopes.

#### Multi-epitope vaccine construction

The particular LBL, CTL, and HTL epitopes of the selected proteins were used to create a vaccine candidate. We linked the chosen HTL, CTL, and LBL employing AAY and KK linkers. These linkers enhance Immunity and epitope display, influencing binding capacity and durability of antigens. The HEYGAEALERAG linker enhances processed by the immune system and suppresses the development of "junctional epitopes." The HEYGAEALERAG linker was included to serve as an interface among CTL, HTL, and LBL epitopes.

#### Evaluation of the physical-chemical Properties of multi-epitope vaccine

The Physical-chemical Properties of the vaccine construct were evaluated using the ProtParam prediction tool (https://web.expasy.org/protparam/). The properties included conceptual isoelectric point (pI), half-life (both *in vitro* and *in vivo*), aliphatic index (AI), instability index (II), molecular weight (MW), and grand average hydropathicity (GRAVY). The Vaxijen v2.0 website was utilized to forecast antigenicity. The AlgPred website (http://crdd.osdd.net/raghava/algpred/) was utilized to assess the allergenic properties of the vaccine candidate, anticipating no adverse reactions. The 3D model of the vaccine structure was designed by SWISS-MODEL (https://swissmodel.expasy.org/) and its Ramachandran diagram was obtained. Verify 3D (https://www.doe-mbi.ucla.edu/verify3d/) was utilized to evaluate the quality of the tertiary model.

#### Examinations of docking protocols

Molecular docking studies may elucidate the chemical relationship between antigen simulations and receptor. The Haddock 2.4 website (https://rascar.science.uu.nl/haddock2.4/) was utilized to submit the enhanced vaccine candidate and the chicken-TLR15 (chTLR15) molecule, therefore establishing the chTLR15 as an immunological receptor. The chTLR15 was selected with PDB ID: 7YLF (PDB DOI: https://doi.org/10.2210/pdb7YLF/pdb). The first phase of receptor formation included the extraction of ligands from amino acids, followed by the elimination of water and other chemical substances. The aforementioned steps were executed with the PyMOL v2.3.4 software. The binding interactions and residues in the contact region were examined. Then, The HawkDock website (http://cadd.zju.edu.cn/hawkdock/) was used to carry out the docking process.

#### In silico cloning of vaccine candidate

The Wrangler (https://www.mrc-lmb.cam.ac.uk/ms/methods/codon.html) prediction tool was utilized to enhance protein synthesis in the animal model. The vaccine candidate construct's sequence was created in the pNZ8121 using the RF-Cloning technique (https://rf-cloning.org/).

### *In vitro* and *in vivo* analysis

#### Bacteria, and vaccine formulation

*Escherichia coli* Top10F and *Lactococcus lactis* PTCC1336 microorganisms were obtained from the Iranian Biological Resource Center for this study. *Lactococcus lactis* bacteria were cultivated in M17 broth manufactured by ZistYar Sanaat CO (ZYS group, Tehran, Iran; https://zys-group.ir/en/). *Escherichia coli* species Top 10F was cultivated in LB broth (ZYS group, Tehran, Iran) at 37°C employing a shaker incubator set to 200 rpm. Merck's microbiological agar medium was included into each of the media at a concentration of 1.5 % to formulate agar media in the plates. The poultry strain of *Salmonella Typhimurium* (ST) was first obtained from the ZistYar Sanat CO. (ZYS group, Tehran, Iran).

#### Construction of L. lactis/Multi epitope

The MoBiTec webpage (https://www.mobitec.com/products/vector-systems/lactococcus-expression-systems/vs-elv00650-01/nice-pnz8121-lactococcus-lactis-secretion-vector-sp-prtp/ecorv#) was utilized to get information on the nisin-based expression vector pNZ8121, while Gene Runner was employed to select the restriction enzyme cleavage sites. The expression vector pNZ8121 is a genetic instrument used in gram-positive bacteria, especially lactic acid bacteria. It comprises critical components for replication inside the host cell, particularly a gene conferring resistance to chloramphenicol. Furthermore, it incorporates a nisin-inducible promoter (PnisA) to regulate gene expression. This expression vector has a total of 3,278 base pairs. The final construct was synthesized employing the 2106 bp multi-epitope gene segment. The gene was ultimately synthetically cloned into the pNZ8121 at the *Kpn*I and *Xba*I restriction sites by Generay, China.

#### Confirmation of multi epitope gene cloning in pNZ8121 vector

The accuracy of the cloning was verified using polymerase chain reaction (PCR), and sequencing methods. The polymerase chain reaction of the multi-epitope DNA was performed employing specified primers, and the resulting amplicon was subjected to electrophoresis on a 1 % agarose gel. GENEray company (GENEray, China) conducted sequencing of the multi-epitope recombinant vector.

#### Transformation

The electroporation approach effectively facilitated the transformation of the recombinant plasmid pNZ8121-Multi epitope into the L*. lactis*. In summary, using an electroporator device (Xcell BIO-RAD Gene Pulser; set to 2500 volts, 25 microfarads, and 200 ohms resistance), 400μl of L*. lactis* were transformed with 6μl (0.2 μg/μl) of the pNZ8121-Multi epitope and 6μl (0.2 μg/μl) of the free pNZ8121 vector. A L*. lactis* suspension was cultivated on an M17 agar media supplemented with chloramphenicol at a concentration of 25 μg/ml. The sample was heated for 72 h at 30°C, with a L*. lactis* suspension (negative control). The chloramphenicol-resistant gene in pNZ8121 was utilized to detect the positively transformed L*. lactis*/pNZ8121-Multi epitope, which was subsequently detected employing colony PCR employing the specified primers (Table 1). The PCR protocol was executed under optimum conditions, including preheating at 94°C for 5 min, 35 cycles of denaturation at 94°C for 40 s, annealing at 58°C for 60 s, and elongation at 72°C for 60 s, concluding with a final step at 72°C for 10 min.

**Table 1:** Primers used in this study.

Gene	Sequence (3′→5′)	TM (°C)	Size (bp)	Accession	Ref
*IFNγ*	F: CTTCATCTGCCTGTGAGTGG R: TCTGATGAACCGCTGAAAAA	60	184	DQ983323.1	This study
*NFkB1α*	F: AGAGGATGCTTCGTTGTGCT R: TCCTGGACAGCAGTGAGATG	60	186	NM_001396396.1	This study
*GAPDH*	F: CAACATCAAATGGGCAGATG R: AGCTGAGGGAGCTGAGATGA	60	130	NM_204305.2	This study
IFN-γ: Interferon gamma NFkB1α: nuclear factor-kappa-B-inhibitor alpha *GAPDH*: Glyceraldehyde 3-phosphate dehydrogenase					

### In-vivo analysis

#### Generation of recombinant L. lactis cells for vaccination purposes

L*. lactis*, Free L*. lactis*/pNZ8121 (retaining the null plasmid as a control group) and L*. lactis*/pNZ8121-Multi epitope were cultivated in M17 media supplemented with a glucose concentration of 1 % and 5 micrograms per milliliter of chloramphenicol (sourced from Sigma-Aldrich) at 30 degrees Celsius with no agitation. Following another centrifugation at 4000 g at 4°C and being rinsed with sterilized ice-cold PBS, the collected cells were suspended again in a vaccination buffer that contains 5 % casein, and 0.5 % glucose and 0.2 M sodium bicarbonate at a concentration of 3 × 10^9^ colony-forming units per milliliter.

#### Experimental animals

One-day-old RAS broilers were obtained from Naghme Toorang Talai CO (https://www.ntorang.com) (Tehran, Iran). The absence of *Salmonella* was verified when arrived by culturing cloacal samples of swabs on XLD agar plates. Chickens were given a diet of a blend of maize and soybeans, free from antibiotics, with water available ad libitum. Furthermore, the hens were exposed to illumination for 15-18 h daily.

#### Experimental design

We purchased 80 one-day-old broiler chickens from Naghme Toorang Talai CO that were clear of Salmonella. The vaccinations began after the chicks were three days old, and the chickens were divided into four groups randomly (Table 2). The avian subjects underwent vaccination with 3 × 10^9^ CFU/100 ml of L*. lactis*, Free L*. lactis*/pNZ8121 (harboring the empty vector, as control) and L*. lactis*/pNZ8121-Multi epitope vaccine orally at day 3 and boosted at 3 week. According to the manufacturer's recommendations, a positive control commercial live vaccine was sprayed on birds that were three days old and supplemented via water consumption at three weeks of age. The poultry were gathered at 3 and 5 weeks of age to obtain blood and cloacal sampling specimens. The birds in the other groups, with the exception of the sham control, were given a fixed dosage of S. typhimurium (3 × 10^9^ CFU/bird) orally at 5 weeks of age, and they were put down 10 days later, as previously mentioned (Han et, al.,2020). On the third day (the day of the main vaccination), the third week (the day of the booster injection), and the fifth week (the day of the challenge), blood and cloacal swabbing specimens were taken from the birds. Serum and cloacal swab samples were obtained during the necropsy. The serum aliquots and cloacal swabs were preserved at -20°C until analysis for antibody responses. Serum and cloacal fluid samples were obtained at the time of necropsy. The cloacal fluid and serum samples were preserved at -20°C prior to antibody testing.

**Table 2:** The number of chickens used in this experiment.

Group	Injection composition	Number of chickens	Type of injection	Volume(µl)	Concentration (CFU/100 ml)
1	L*. lactis*/pNZ8121-Multi epitope	20	oral	100 µl	3 × 10^9^ CFU/100ml
2	Free L*. lactis*/pNZ8121	20	oral	100 µl	3 × 10^9^ CFU/100ml
3	L*.lactis*	20	oral	100 µl	3 × 10^9^ CFU/100ml
4	PBS	20	oral	100 µl	100 µl/—
5	Commercial	20	oral	According to data sheets	

#### Enzyme-linked immunosorbent assay (ELISA)

The detection of antigen-specific antibodies was the focus of the development of the ELISA. In summary, 96-well plates were coated with pre titrated Multi epitope proteins utilizing a carbonate-bicarbonate buffer (pH 9.6) at 4°C for 24 h. Utilizing 5 % (v/v) skim milk (Parsilact, Iran) dissolved in PBS, the support surfaces had been blocked for a single hour at 37°C. A one-hour incubation period was conducted at 37°C for the prediluted serum (1:800), cloacal swab (1:1), small intestine wash (1:4), and bile (1:800) samples after they were put into the correct triplicate wells. Following three rounds of washing, pre-titrated goat anti-chicken IgA-HRP (1:3,000) or IgY-HRP (1:10,000) (Gallus Immunotech, Shirley, MA) was applied to the plate. The 3,30,5,50-Tetramethylbenzidine (TMB) (Sigma-Aldrich, USA) reagent was introduced after an hour of incubation at 37°C, and the reaction was halted by adding 1 M phosphoric acid. An optical density (OD) reader (Molecular Devices, CA) was used to measure the readings at 450 nm.

#### Real-time PCR assay

The animals were given anaesthesia, and then the spleen and small intestine were removed and kept in a nitrogen-based liquid at -198°C. Following that, tissue samples' RNA was isolated and transformed into cDNA using YTA kits made in Iran by Yekta Tajhiz. The *GAPDH* gene served as an internal standard, and the real-time PCR was conducted using the YTA master mix (SYBR green, Yekta Tajhiz, Iran). Real-time PCR was carried out utilizing a 15μL reaction mixture that contained 10μL of master mix, 3.5μL of double sterile distillate water, 0.5μL of cDNA, 0.5μL of forward primer, and 0.5μL of reverse primer. There were 40 cycles in the temperature-dependent cycle program, with 20 s at 95°C and 40 s at 60°C. A first denaturation process lasting ten minutes at 95°C came before this. The relative gene transcription levels of interferon-gamma (IFNγ), and nuclear factor-kappa-B-inhibitor alpha (NFkB1α) were assessed using the 2^−ΔΔCT^ technique. The *GAPDH* levels in each sample were then compared to these values for standardization.

### Statistical analyses

Data processing and statistical assessment was performed utilizing GraphPad Prism 5.0. The findings of a one-way analysis of variance (ANOVA) comparing the median scores were evaluated using Tukey-Kramer research. One tailed unpaired t-test was used to analyses 2 samples. A 95 % confidence interval was used in the assessment process. There were notable variations at *p* < 0.05 or *p* < 0.01.

## Result

### *In silico* analyses

#### Screening was done on outer membrane antigens

Fifteen antigens in the extracellular layer or outside membrane were found and chosen based on research from the Vaxign website. After outer membrane proteins (OMP) and extracellular components were identified, the accession number for the specified antigens was found on the NCBI website. The protein screening process was prolonged based on criteria such as non-allergenicity and antigenicity more than 0.5. The resulting information was used to test six proteins for *Salmonella Enteritidis* and six proteins for *Salmonella Typhimurium* (Table 3).

**Table 3:** Screening of *Salmonella Enteritidis* and *Salmonella Typhimurium* based on reverse vaccinology.

Pathogen	Protein profile		Localization		Antigenicity	Allergenicity
	**Protein name**	**GenBank**	**Type**	**Reliability**	**VaxiJen**	**Algpred**
*Salmonella Enteritidis*	outer membrane protein	AAD51878.1	Outer Membrane	4.212 *	0.7723	non-allergenic
	surface-exposed virulence protein BigA	ELY02691.1	Outer Membrane	4.212 *	0.8301	non-allergenic
	flagellin	2108321A	Extracellular	4.735 *	0.8045	non-allergenic
	Large extracellular alpha-helical protein	KOX82698.1	Outer Membrane	1.866 *	0.5523	non-allergenic
	curli production assembly component CsgF	OPP83819.1	Extracellular	2.658 *	0.6214	non-allergenic
	porin	ELO89407.1	Extracellular	2.250 *	0.9482	non-allergenic
*Salmonella Typhimurium*	outer membrane protein	ACO71192.1	Outer Membrane	4.168 *	0.7835	non-allergenic
	outer membrane protein AsmA	ASF64994.1	Outer Membrane	1.648 *	0.7356	non-allergenic
	type VI secretion system tube protein Hcp	QPD99836.1	Extracellular	1.028 *	0.5020	non-allergenic
	flagellin	AAB33953.1	Extracellular	4.149 *	0.7795	non-allergenic
	vsdB protein	S15214	Extracellular	2.726 *	0.5690	non-allergenic
	opacity protein-like surface antigen	PZV93137.1	Outer Membrane	4.031 *	0.5759	non-allergenic
Threshold				* More than 1	More than 0.5	—

#### B-cell and T-cell epitopes forecasting

Analysis of the ABCpred website showed that the vaccine candidate had linear B-cell epitopes with a cutoff score above 0.90. Linear B-cell epitopes with rank 1 were used for subsequent analyses (Table 4). Analysis of the NetCTL v1.2 website showed that the vaccine candidate had CTL epitopes with a cutoff score above 0.90. CTL epitopes with a rank of 1 were used for subsequent analyses. Analysis of the NetMHCIIpan v. 3.2 website showed that CTL epitopes with a cutoff score greater than 0.89 were present in the vaccination candidate. For further analysis, HTL epitopes with scores greater than 0.89 for the DRB1×01:01, DRB1×03:01, DRB1×09:01, DRB1×11:01, and DRB1×15:01 alleles were used (Table 4). Supplementary Material 2 shows the full details of all screening of LBL, CTL, and HTL epitopes.

**Table 4:** Screening of *Salmonella Enteritidis* and *Salmonella Typhimurium* LBL, CTL, and HTL epitopes.

Pathogen	Protein name	LBL			CTL			HTL		
		**Rank**	**Sequence**	**Score**	**Rank**	**Sequence**	**Score**	**Allele**	**Core Sequence**	**Score**
*Salmonella Enteritidis*	**outer membrane protein**	1	AASIGAAVAFMPFATQ	0.94	1	KTDIWDYDM	1.4919	DRB1_0101	FQNMTGNDG	0.963538
		2	DMIGQAPGNGINGDYD	0.93	2	AVFDWDNHY	1.3282	DRB1_0301	IYYDVVGVK	0.856390
		3	GVGITFQLDYGFRVDY	0.92	3	KSIGNVDLY	1.2600	DRB1_0901	FRFAADYYL	0.808463
		4	GDRIEYVNGRDYLRTD	0.91	4	FDDISWISY	1.1993	DRB1_1101	FMTTKKVSV	0.671496
		5	PAVFDWDNHYAKGATD	0.90	5	FSDDYNANN	1.0746	DRB1_1501	VFDWDNHYA	0.840871
	**surface-exposed virulence protein BigA**	1	SGTITATNGYSAITTA	0.98	1	ASQNLTLEY	3.1573	DRB1_0101	FSSATGVSV	0.967445
		2	SNHSYTPPTPDNGGDD	0.96	2	TADGVINVY	2.6931	DRB1_0301	VAEDGKSLV	0.934350
		3	AMGIITYGTGNEAKNT	0.94	3	FTAGTLANY	2.5654	DRB1_0901	FSSATGVSV	0.911304
		4	LKHSMAFDEGLAWNNS	0.94	4	VLEGAVWNY	2.3222	DRB1_1101	IINLTRAND	0.846358
		5	TGSIAGTSYQQEIVNT	0.94	5	DTALHLDAY	2.1894	DRB1_1501	VTPYAGVKF	0.893249
	**flagellin**	1	TFTIDTKTGDDGNGKV	0.95	1	YTSVVNGQF	2.1221	DRB1_0101	LINEDAAAA	0.839374
		2	RGAIKGGKEGDTFDYK	0.93	2	SSFKNVTGY	1.5381	DRB1_0301	FTIDTKTGD	0.836513
		3	DGISIAQTTEGALNEI	0.88	3	ATDVNAATL	1.4628	DRB1_0901	YVNAANGQL	0.764203
		4	RSRIEDADYATEVSNM	0.88	4	TLQSSKNVY	1.0874	DRB1_1101	FTSNIKGLT	0.849839
		5	GKEGDTFDYKGVTFTI	0.86	5	RSRIEDADY	0.9653	DRB1_1501	VTGYDTYAA	0.947044
	**Large extracellular alpha-helical protein**	1	RVMAQAWTADDFGRGE	0.93	1	VSQAENGLY	3.0565	DRB1_0101	IIAQQAQPI	0.966630
		2	VESSDGPLWWQAIDVP	0.93	2	ASFVSQWEY	2.6992	DRB1_0301	LKLDKASYR	0.961222
		3	GESWHVPEQHLANVSP	0.93	3	TSGLFPALY	2.4845	DRB1_0901	YNYSNAATL	0.924826
		4	TGKVSFPVEWGAYRLE	0.92	4	FSVVGYYLY	2.4565	DRB1_1101	YAPQWRATG	0.931620
		5	LKMADLVYTGRFDLNP	0.92	5	FAAKAVYDY	1.9549	DRB1_1501	IADYGSSLR	0.708347
	**CsgF**	1	TQAIQSQILGGLLTNI	0.91	1	NSAQAQNSY	2.7530	DRB1_0101	FGIETPSAL	0.735311
		2	DFGIETPSALDNFTQA	0.87	2	VTDRKTGRT	0.7665	DRB1_0301	FIIDIANRD	0.738534
		3	TGKPGRMVTNDFIIDI	0.85	3	ALDNFTQAI	0.7567	DRB1_0901	FTQAIQSQI	0.674487
		4	RKTGRTSTIEVSGLQT	0.85	4	VTNDFIIDI	0.7135	DRB1_1101	LNVTDRKTG	0.894506
		5	PNFGGNPNNGSFLLNS	0.84	5	LTWAGNMTF	0.7029	DRB1_1501	IIDIANRDG	0.260039
	**porin**	1	HTMEDGYKERSAGDFN	0.93	1	DTALHLDAY	2.1894	DRB1_0101	INGLATIGV	0.825185
		2	GWSATATLEGGPNLSY	0.92	2	GLATIGVKY	1.2268	DRB1_0301	LKLDYAGKD	0.283963
		3	YSSNDTALHLDAYQWK	0.90	3	YSSNDTALH	1.1074	DRB1_0901	WSATATLEG	0.699558
		4	NGLATIGVKYSSNDTA	0.89	4	SSNDTALHL	0.8774	DRB1_1101	YSKSQRTAS	0.665481
		5	KVTPYAGVKFRHTMED	0.87	5	SIVGLKLDY	0.8495	DRB1_1501	VTPYAGVKF	0.893249
*Salmonella Typhimurium*	**outer membrane protein**	1	DMIGQAPGNGINGDYD	0.93	1	AGDAWGLEY	2.0067	DRB1_0101	FQNMTGNDG	0.963538
		2	AASIGAAMAFMPFATQ	0.92	2	LTWGTAWNY	1.9269	DRB1_0301	IYYDVVGVK	0.856390
		3	GVGITFQLDYGFRVDY	0.92	3	LTDDLTWGT	1.8225	DRB1_0901	FRFAADYYL	0.808463
		4	GDRIEYVNGRDYLRTD	0.91	4	KTDIWDYDM	1.4919	DRB1_1101	FMTTKKVSV	0.671496
		5	TVLKQDPQAGNPLSRL	0.88	5	ASYLFSDDY	1.4816	DRB1_1501	VDLYASYLF	0.840505
	**outer membrane protein AsmA**	1	DGEMSLPGTLDARTAS	0.94	1	GTILKAFNY	1.3572	DRB1_0101	YQGLQSFTA	0.970114
		2	ARTASPRIEFHPRLNH	0.93	2	LTTLMILLV	1.1388	DRB1_0301	VRSDDAPVA	0.853875
		3	KAHVDMSNTRLEGMNF	0.92	3	HVDMSNTRL	1.0520	DRB1_0901	YQGLQSFTA	0.702732
		4	GGDAQQSQENMDNATR	0.88	4	VAEDRGWSF	0.8641	DRB1_1101	FRHSWKGKA	0.883134
		5	SAVAQTGGAVSQGQNT	0.88	5	RADNMRLDV	0.8184	DRB1_1501	FRAYMVQQV	0.770369
	**type VI secretion system tube protein Hcp**	1	HLTAMHECRYNFCLNS	0.95	1	YLNYYTNIY	2.8162	DRB1_0101	YNRLFPATL	0.925792
		2	DIYIEGIGTENNKADS	0.95	2	LTAMHECRY	2.7237	DRB1_0301	FEQDPVLVR	0.901563
		3	KKTGFDGTEAGSYLNY	0.94	3	ATAFQPVEY	2.5687	DRB1_0901	FYGTHSLQF	0.849909
		4	LKLIGKYIHCSANWNA	0.93	4	ISQAKNAFY	2.4958	DRB1_1101	YHIEVRGAG	0.980523
		5	EGCGSEPSVGNRYQTG	0.93	5	MSDIIYLKI	2.1143	DRB1_1501	ITIHYDYIR	0.519764
	**flagellin**	1	ALAQVDTLRSDLGAVQ	0.92	1	SISINTTKY	1.8404	DRB1_0101	LQKIDAALA	0.890933
		2	ASVVKMSYTDNNGKTI	0.92	2	TASVVKMSY	1.5558	DRB1_0301	IDIDLKQIN	0.939459
		3	TYAASKAEGHNFKAQP	0.90	3	DTLNVQQKY	1.4815	DRB1_0901	YKVSDNVQV	0.813172
		4	TKYTADDGTSKTALNK	0.89	4	YTDNNGKTI	1.4534	DRB1_1101	LNEIDRVSG	0.909246
		5	GKTIDGGLAVKVGDDY	0.89	5	RSRIEDSDY	1.0917	DRB1_1501	LKQINSQTL	0.711736
	**vsdB protein**	1	LGLKTADYTPFEALNT	0.96	1	TATDLTSFY	2.1130	DRB1_0101	YAMELASRL	0.981007
		2	RVRGTLQQTPDNGTNL	0.93	2	SLTGDNSNY	1.1222	DRB1_0301	VVNDAINQQ	0.601098
		3	PEQQTLPTEPDNSTAT	0.93	3	ATDLTSFYQ	1.0108	DRB1_0901	YAMELASRL	0.719166
		4	VGGIQGQAERRPDLAT	0.92	4	NTDVSLEDI	0.9574	DRB1_1101	VETAIRAPA	0.656779
		5	TVPPGGTVDCGYSACQ	0.89	5	STATDLTSF	0.9395	DRB1_1501	YNYYSVFDI	0.371726
	**opacity protein-like surface antigen**	1	FGYAQTHLSSLKNSDS	0.92	1	HSKEDSFAY	1.8336	DRB1_0101	YVSLYGNAG	0.983121
		2	AGVIFNPVKSISIDAS	0.91	2	SVDYGSLMF	1.7865	DRB1_0301	LANDQHTVS	0.545816
		3	IDASWETSRFFAVDTN	0.90	3	ATRNEMENY	1.6536	DRB1_0901	FNPVKSISI	0.906304
		4	YGSLMFGPTYRFNDYV	0.89	4	NTFGVSVGY	1.3134	DRB1_1101	YVSLYGNAG	0.477149
		5	NETWGMLGSFTATRNE	0.89	5	GSLMFGPTY	1.0834	DRB1_1501	VSLYGNAGI	0.940070
Threshold		More than 0.90			More than 0.90			More than 0.89		

#### Constructing of the multi-epitope vaccine

**Fig. 1:**
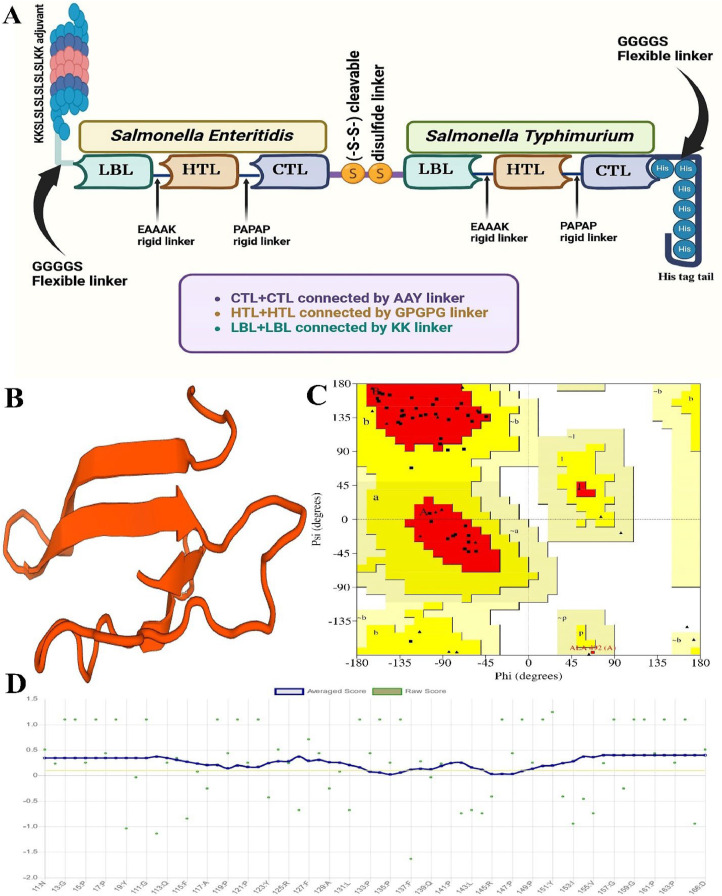
**A)** The overall design protocol of vaccine. **B)** The 3D structure of the vaccine structure was designed by SWISS-MODEL. **C)** the Ramachandran plot indicated that 90.9 % of the residues were located in the preferred zone. **D)** At least 80 % of the amino acids have scored ≥ 0.1 in the 3D/1D profile.

The HTL, CTL, and LBL epitopes were the targets of the multi-epitope vaccine candidate. AAY, GPGPG, and KK linkers connected the CTL, HTL, and LBL epitopes, respectively. The *Salmonella Enteritidis* and *Salmonella Typhimurium* fusion peptides were joined using the SS (-S-S-) cleavable disulfide linker. The HIS×6 tail was attached to the end of vaccine constructs. To increase the immunogenicity of the vaccine design, an adjuvant, KKSLSLSLSLSLSLKK, was added at the N-terminal (Pogostin et al., 2022). The overall design protocol of this vaccine was shown in Fig. 1A. Additionally, Table 5 displayed the sequence of vaccine candidate.

**Table 5:** The structure displays unique characteristics in terms of its antigenic properties, allergy, and physicochemical properties.

MKKSLSLSLSLSLSLKKGGGGSAASIGAAVAFMPFATQKKSGTITATNGYSAITTAKKTFTIDTKTGDDGNGKVKKRVMAQAWTADDFGRGEKKTQAIQSQILGGLLTNIKKHTMEDGYKERSAGDFNEAAAKFQNMTGNDGGPGPGFSSATGVSVGPGPGVAEDGKSLVGPGPGFSSATGVSVGPGPGVTGYDYAAGPGPGIIAQQAQPIGPGPGLKLDKASYRGPGPGYNYSNAATLGPGPGYAPQWRATGGPGPGLNVTDRKTGGPGPGVTPYAGVKFPAPAPKTDIWDYDMAAYASQNLTLEYAAYYTSVVNGQFAAYVSQAENGLYAAYNSAQAQNSYAAYDTALHLDAYSSDMIGQAPGNGINGDYDKKDGEMSLPGTLDARTASKKHLTAMHECRYNFCLNSKKALAQVDTLRSDLGAVQKKLGLKTADYTPFEALNTKKFGYAQTHLSSLKNSDSEAAAKFQNMTGNDGGPGPGYQGLQSFTAGPGPGYNRLFPATLGPGPGFEQDPVLVRGPGPGYHIEVRGAGGPGPGIDIDLKQINGPGPGLNEIDRVSGGPGPGYAMELASRLGPGPGYVSLYGNAGGPGPGFNPVKSISIGPGPGVSLYGNAGIPAPAPAGDAWGLEYAAYGTILKAFNYAAYYLNYYTNIYAAYSISINTTKYAAYTATDLTSFYAAYHSKEDSFAYGGGGSHHHHHH			

Row	**Characteristics**	**Finding**	**Remark**
1	Number of amino acids	571	Suitable
2	Molecular weight (Da)	72277.34	Average
3	Theoretical pI	8.62	alkaline
4	Chemical formula	C_3215_H_4892_N_872_O_1000_S_13_	—
5	Instability index of vaccine	27.06	Stable
6	Aliphatic index of vaccine	61.32	Thermostable
7	GRAVY	-0.405	Hydrophilic
8	Antigenicity	0.9533	Antigenic
9	Immunogenicity	Positive	Immunogenic
10	Allergenicity	No	Non-allergen

#### The physicochemical characteristics of multi-epitope vaccine

It has been shown that the multi-epitope vaccine, which has a molecular weight of 72277.34 Da, is non-allergic and immunogenic (Ag score: 0.9533). A projected pI of 8.62 and an instability score of 26.82 indicate that the vaccine design was stable. The vaccine had a half-life of 30 h *in vitro* in mammalian reticulocytes but only around 20 h in yeast and 10 h in *E. Coli in vivo*. Anticipated values for the secondary structure of the vaccine construct were a GRAVY value of -0.405 and an Aliphatic index of 61.24 % (Table 5). The anticipated secondary configuration of the vaccine construct comprised The total quantity of residues that are negatively charged (Asp + Glu) of 51 and a total quantity of residues that are positively charged (Arg + Lys) of 56.

#### Multi-epitope vaccine construct modeling, refining, and validation

The 3D structure of the vaccine structure was designed by SWISS-MODEL (Fig. 1B). and its Ramachandran diagram was obtained (Table 5B). Moreover, the study of the Ramachandran plot indicated that 90.9 % of the residues were located in the preferred zone (Ramachandran Favored=90.9 %), residues in liberally permitted areas 3 %, residues in extra permitted locations 6.1 %, Bad Bonds (0/480), Bad Angles (7/657, A541 ASP, (A481 GLY-A482 PRO), (A495 GLY-A496 PRO), A499 ASN, A527 HIS, A512 PHE) (Fig. 1C). The Overall Quality Factor of vaccine was 72.4138. According to results 87.88 % of the residues have mean 3D-1D score ≥ 0.1. In fact, The 3D/1D profile gives at least 80 % of the amino acids with a score of ≥ 0.1 (Fig. 1D).

#### Multi-epitope vaccine construct and TLR15 complex have been subjected to molecular docking

**Fig. 2:**
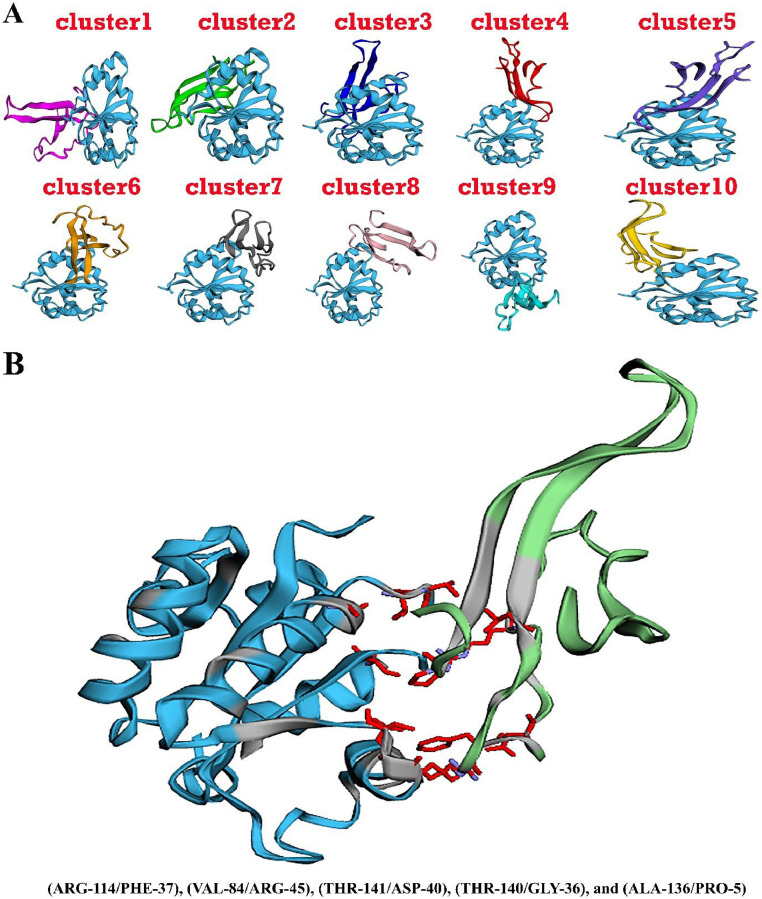
**A)** Alignment of the TOP 10 structure of the selected model of the designed vaccine and the TLR-4 receptor. B) The multi epitope vaccine was subjected to molecular docking with the immunological receptors TLR15. The molecular docking outcomes of the most favorable associations, namely (ARG-114/PHE-37), (VAL-84/ARG-45), (THR-141/ASP-40), (THR-140/GLY-36), and (ALA-136/PRO-5), were shown.

Establishing meaningful contact between the immune cell and the vaccine design is important for achieving a robust and consistent immune response. The Cluspro software generates 10 distinct clusters, each characterized by elevated interaction energies (Table 6). The score of the top five clusters were -3031.54, -2942.89, -2829.05, -2812.96, and -2728.34. The first cluster was chosen based on its superior energy score (-3031.54) compared to the other clusters (Fig. 2A). Also, interaction atoms of the top molecular docking model were shown in Fig. 2B. The first vaccine model was chosen as the optimal model. In addition, the binding free energy of the optimal complex was determined to be **-20.77** (kcal/mol). Fig. 2B illustrates the three-dimensional structural alignment between the chosen model of the developed vaccine and the TLR-15 receptor. The molecular docking outcomes of the most favorable associations, namely (ARG-114/PHE-37), (VAL-84/ARG-45), (THR-141/ASP-40), (THR-140/GLY-36), and (ALA-136/PRO-5), were shown.

**Table 6:** Summary of the top 10 models of Vaccine construct and TLR15 complex Docking result.

Rank	Score	Binding free energy of complex (kcal/mol)	Docking result link
1	-3031.54	**-20.77**	http://cadd.zju.edu.cn/hawkdock/result/VAC_model1-1729451765585
2	-2942.89	**-20.91**	http://cadd.zju.edu.cn/hawkdock/result/VAC_model2-1729453025463
3	-2829.05	**-26.76**	http://cadd.zju.edu.cn/hawkdock/result/VAC_model3-1729453113941
4	-2812.96	**-25.26**	http://cadd.zju.edu.cn/hawkdock/result/VAC_model4-1729453135526
5	-2728.34	**-34.71**	http://cadd.zju.edu.cn/hawkdock/result/VAC_model5-1729453154561
6	-2701.03	**-25.74**	http://cadd.zju.edu.cn/hawkdock/result/VAC_model6-1729453187550
7	-2688.21	**-28.26**	http://cadd.zju.edu.cn/hawkdock/result/VAC_model7-1729453227941
8	-2656.24	**-15.85**	http://cadd.zju.edu.cn/hawkdock/result/VAC_model8-1729453243734
9	-2171.04	**-34.11**	http://cadd.zju.edu.cn/hawkdock/result/VAC_model9-1729453339790
10	-1906.50	**-24.02**	http://cadd.zju.edu.cn/hawkdock/result/VAC_model10-1729453357014

#### Optimization of multi-epitope vaccines and in-silico cloning

The Codon optimization Tool was used to enhance the codon utilization of the immunization, resulting in an optimal codon sequence of 2106 nucleotides. With a GC level of 49.38 %, the optimized sequences suggested that the host cell (*Lactococcus lactis*) may produce the vaccine construct. The restriction patterns of the restriction enzymes *Xho*I and *Not*I were then added to the N and C terminals of the altered codon sequence. Furthermore, the altered DNA was cloned into the pNZ8121 vector using the SnapGene software.

### Results of *in vitro* studies

#### Generating and identifying recombinant multi-epitope vaccine

**Fig. 3:**
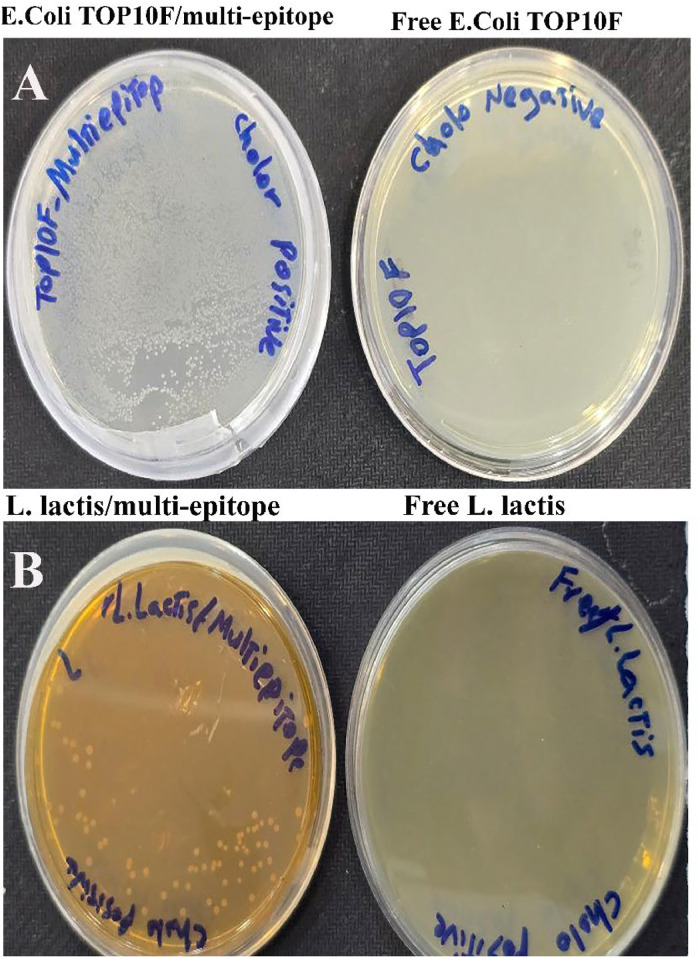
**A)** The process of recombinant vector transformation was verified by comparing individual colonies cultivated in the medium containing antibiotic with the negative control group, which consisted of bacteria without the recombinant vector. **B)** The process of electroporation of multi-epitope gene was successfully performed in L*. lactis****.*** The existence of the recombinant plasmid was confirmed using the presence of bacterial colony on MRS agar supplemented by specific antibiotic.

The DNA vaccine construct was created by inserting the multi-epitope multi-epitope gene into the expression vector pNZ8121. The restriction enzymes *Kpn*I and *Xba*I were used to digest the plasmid. The extraction of the digestion segments at 2106 bp using electrophoresis confirmed the formation of the recombinant plasmid. To preserve and replicate the plasmid encoding the multi-epitope gene, the transformation procedure was performed in the *E. Coli* strain TOP10. The individual colonies that were obtained were used to create matrices. Compared to the negative control group, which included *E. Coli* strain TOP10 bacteria without the recombinant vector, the transformation was verified by individual colonies grown in culture conditions treated with the antibiotic chloramphenicol. Following the transformation, the produced matrices were placed in an LB-Broth medium that had been treated with chloramphenicol. Also, the plate of *E. Coli* TOP10F bacteria transformed with multi-epitope gene was shown in Fig. 3B (Fig. 3A).

#### Results of creation of recombinant L. lactis with multi-epitope gene (L. lactis/multi-epitope) as live bacterial vector

Electroporation was used to insert the recombinant plasmid carrying the vaccine multi-epitope gene into the *Lactococcus lactis* bacterium. According to the findings, *Lactococcus lactis* strains that were given the pNZ8121/multi-epitope plasmid were able to proliferate on M17 culture media containing the antibiotic chloramphenicol (Fig. 3B).

#### Antibody reaction to pathogenic antigens following vaccination

**Fig. 4:**
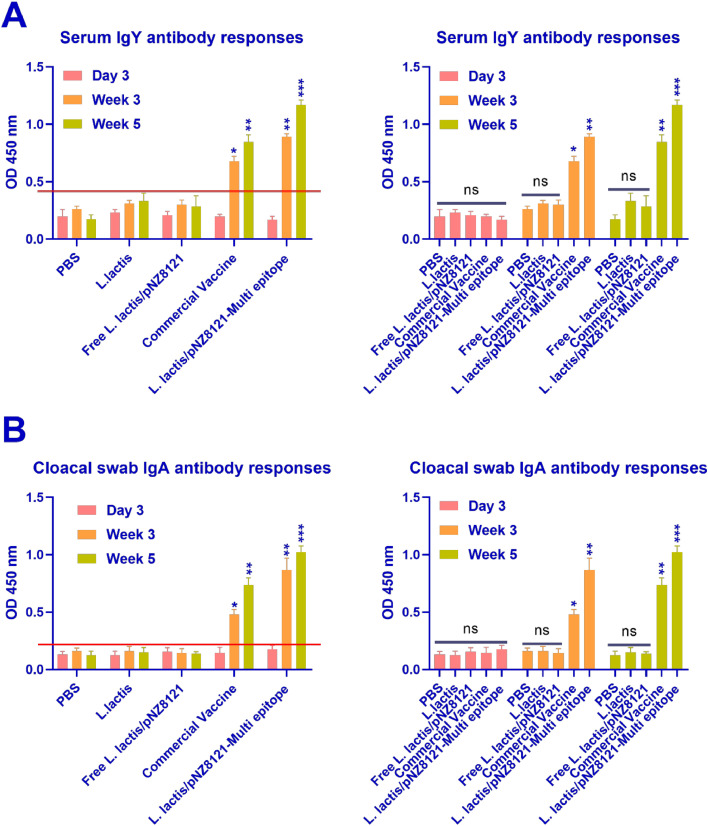
IgY and IgA antibody reactions to particular multi-epitope antigens after immunization. A separate vaccination was given orally to broilers at age 3 days, and they received booster doses at ages 3 weeks and 5 weeks. A commercial Commercial live *Salmonella* vaccine that was administered on the same days as the candidate vaccines served as a control. ELISA was used to test the obtained blood and cloacal swab samples in order to identify particular IgY and IgA antibodies, respectively. (A) The IgY antibody reaction was assessed in serum samples. (B) The IgA antibody reaction was examined in cloacal swabs. The mean±SEM of 10 birds per group is used to show the data. One-way ANOVA and the Tukey post-hoc test was used to identify significant differences between the groups. The Fig.s only show significant differences (*p < 0.05, **p < 0.01, and ***p < 0.001).

After the initial and booster vaccinations were administered, blood and cloacal samples were taken in order to measure the concentrations of vaccine-specific antibody reactions after the vaccination. All study groups did not show a rise in specific IgY and IgA antibody amounts after the first dose of the vaccines (the birds were 3 days old). However, as in comparison with control groups, the serum samples of birds vaccinated with L*. lactis*/pNZ8121-Multi epitope and commercial vaccine showed substantially higher amounts of IgY antibodies (*p* < 0.001 and *p* < 0.01) at 5-week age (Fig. 4A). Furthermore, in comparison to birds in the control group, commercial vaccine and L*. lactis*/pNZ8121-Multi epitope vaccine-inoculated birds exhibited substantially higher levels of anti-Multi epitope IgA antibody (*p* < 0.001and *p* < 0.01) in cloacal swab tests (Fig. 4B).

#### Cytokine gene expression analysis by real-time PCR

**Fig. 5:**
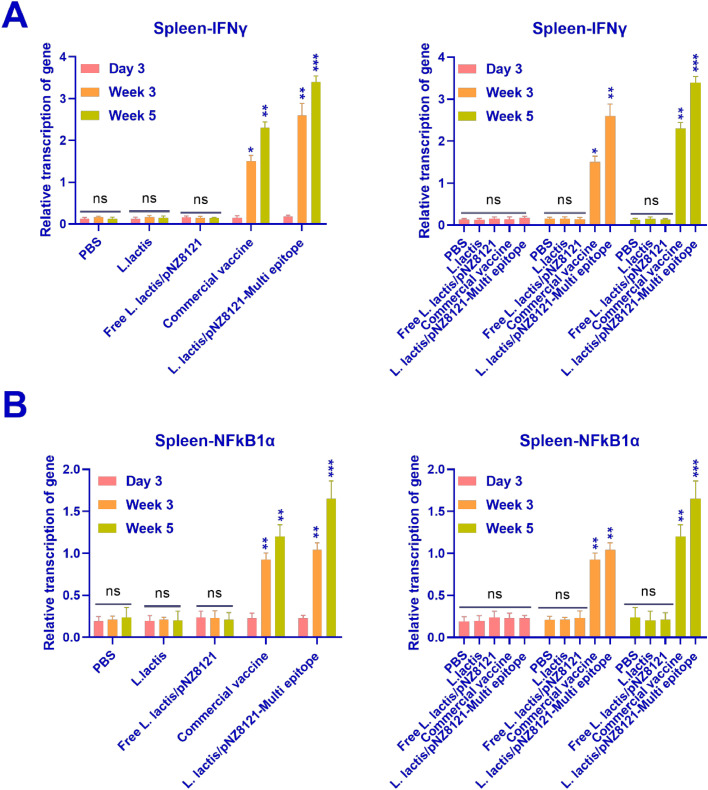
**A)** IFN-γ cytokine concentrations were detected in the spleen of the control and vaccinated groups. The highest amount of transcription was observed in week 5. Also, the L*. lactis*/pNZ8121-Multi epitope group showed the highest amount of transcription in week 5 compared to the other groups. **B**) The amounts of NFkB1α cytokines were detected in the spleen of the control and vaccinated groups. NFkB1α transcription peaked in week five. Additionally, in comparison to the other groups, the L*. lactis*/pNZ8121-Multi epitope group had the greatest level of NFkB1α transcription in week five. *p < 0.05, **p < 0.01, and ***p < 0.001.

**Fig. 6:**
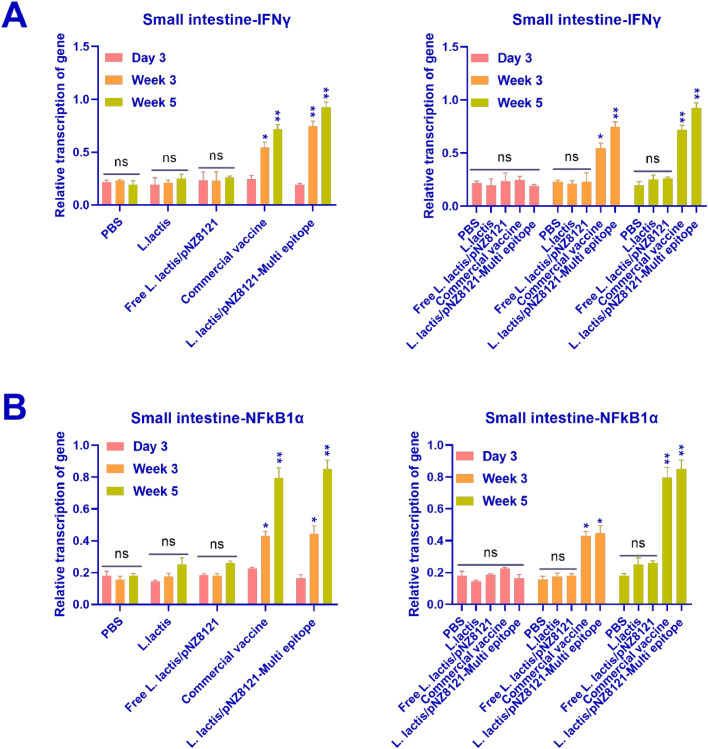
**A)** Both the control and vaccinated groups' small intestine showed levels of the cytokine IFN-γ. In week five, most of the transcription was recorded. Additionally, in comparison to the other groups, the L*. lactis*/pNZ8121-Multi epitope group had the greatest level of transcription in week five. **B)** Both the control and vaccinated groups' small intestine had levels of NFkB1α cytokines. In week five, NFkB1α transcription peaked. Furthermore, the L*. lactis*/pNZ8121-Multi epitope group exhibited the highest amount of NFkB1α transcription in week five when compared to the other groups. *p < 0.05, **p < 0.01, and ***p < 0.001.

Nuclear factor-kappa-B-inhibitor alpha (NFkB1α), and interferon-gamma (IFNγ) transcription rates were measured in the spleen using real-time PCR. The activation of cytokine from chickens immunized with Free L*. lactis*/pNZ8121, L*. lactis*, or PBS resulted in significantly lower concentrations of IFN-γ, and NFkB1α than the stimulation of spleen cytokine in chickens after vaccination with L*. lactis*/pNZ8121-Multi epitope and Commercial vaccine (*p* < 0.001, *p* < 0.01, as shown in Fig. 5A, and 5B). Following immunization with the L*. lactis*/pNZ8121-Multi epitope and Commercial vaccines, the chickens showed activation of IFN-γ, with corresponding values of 3.4 ± 0.17 and 2.3 ± 0.16. Fig. 5 shows the levels of NFkB1α cytokines in the various groups. As demonstrated in Fig. 6A, and 6B, the activation of small intestine cytokine from chickens immunized with Free L*. lactis*/pNZ8121, L*. lactis*, or PBS resulted in significantly lower amounts of IFN-γ, and NFkB1α than the stimulation of small intestine cytokine in animals following vaccination with L*. lactis*/pNZ8121-Multi epitope and Commercial vaccine (Fig. 6A, and 6B).

## Discussion

*Salmonella* uses flagella to attach itself to the mucosal epithelial cell of poultry's small intestines, where it becomes absorbed by mucosal M cells in Peyer's patches (Ijaz et al., 2021). Thus, creating a *Salmonella* vaccine that targets the development of intestinal mucosal immunity may lessen bacterial colonization and spread in sick birds, thus preventing the human spread of the disease (Lillehoj et al., 2003). The underlying processes of vaccine-induced immunity must be understood in order for vaccinations to have the intended effectiveness versus *Salmonella* infection. Through the colonization of live bacterial vectors in mucosal cells in the small intestine, the live bacterial vector-based vaccine delivery platform prevents the entrapped antigens from degrading and promotes their internalization (Lu et al., 2024). Thus, it is known that powerful live bacterial vector-based vaccines administered via the mucosa may produce immune responses similar to those caused by natural mucosal pathogen infections, such as *Salmonella* (Luria-Pérez et al., 2022).

Live bacterial vector-based oral vaccines are very appropriate and provide several advantages (Jiang et al., 2019). These vaccinations work well to elicit mucosal and systemic immune responses. Because frozen stockpiles are not required, they also have the benefit of simplified large-scale manufacture and storage (Harris et al., 2016). These vaccinations are also safer since they do not include any microbial toxins or human pathogenic agents (Jansen et al., 2018). The results of our investigation show that when given orally, L*. lactis*/pNZ8121-Multi epitope, a live bacterial vector, may successfully elicit strong immune responses in birds. The levels of protection attained were often on par with those obtained by commercial vaccine. To combat the *Salmonella* multi-epitope gene, we created a live bacterial vector-based vaccine delivery system that reduces the challenge *Salmonella* burden in the intestines of both broiler-type chickens and produces strong local mucosal immunity. This research assessed the cross-protective effectiveness of the L*. lactis*/pNZ8121-Multi epitope vaccination in broilers. Similar to earlier research (Dolatyabi et al., 2024), oral immunization with prime-boost L*. lactis* in chickens resulted in higher levels of mucosal sIgA specific to the epitope antigen and systemic IgY antibody responses than control vaccine groups. These findings are corroborated by research done on mice, which revealed that immunity could be produced in the animal model by using the L*. lactis* platform as a live bacterial vector.

The absence of suitable and secure adjuvants that act at mucosal sites is a major barrier to the development of live bacterial vector vaccines (Lycke et al., 2012). The concurrent injection of appropriate adjuvants is often necessary for the generation of immune responses after mucosal vaccination. Overcoming resistance and starting the transition from innate to adaptive immunity are the functions of these adjuvants (De Brito et al., 2009). However, a number of adjuvants have been associated with toxicity or adverse effects, and their use significantly increases the cost of vaccine manufacturing and adds complexity (Petrovsky et al., 2004). The plasmid structure's use of KKSLSLSLSLSLSLKK adjuvants has reduced the negative effects of adjuvants while demonstrating little toxicity. The research successfully increased the immune response by using the KKSLSLSLSLSLSLKK adjuvant in the plasmid combination (Han et al., 2020).

The study found that birds who received the L*. lactis*/pNZ8121-Multi epitope vaccination had a considerably higher blood serum concentration of Multi epitope-specific sIgA (*P* < 0.001). Comparing the concentrations to the PBS negative control, they returned to their initial state, although comparable significant differences were still seen. According to the findings, the concentrations of Multi epitope-specific sIgA in the commercial and L*. lactis*/pNZ8121-Multi epitope vaccine groups were significantly higher than those observed in the PBS control group three weeks after the animals had received the L*. lactis*/pNZ8121-Multi epitope vaccination. Compared to the negative control groups, samples from animals that received the L*. lactis*/pNZ8121-Multi epitope and commercial vaccine had considerably greater levels of IFN-γ and NFkB1α (*P* < 0.001). Assessing NFKB1α in poultry infected with Salmonella may provide significant insights into the avian immune reaction to the illness. Activation of NF-κB results in the synthesis of several inflammatory mediators, including cytokines and chemokines, which are crucial for the recruitment of immune cells to the infection site and the regulation of bacterial reproduction (Lycke et al., 2012). A reduction in NFKB1α levels would imply heightened NF-κB activity, indicating a robust inflammatory reaction to Salmonella infection. An elevation in NFKB1α levels may indicate a diminished or inhibited inflammatory reaction, perhaps correlating with a more severe illness. Tracking NFKB1α levels during Salmonella infection may assist researchers in comprehending the temporal variations in the avian immune response. Monitoring NFKB1α in poultry infected with Salmonella may provide significant insights into the avian immune response to the illness. This knowledge may enhance our comprehension of the etiology of Salmonella infection, facilitate the development of superior diagnostic tools, and assess the effectiveness of many therapies designed to manage this significant poultry illness (Dolatyabi et al., 2024).

## Conclusion

Our work offers a fresh approach to developing immunogenic potential multi-epitope gene utilizing the noncommercial, food-grade, and nonpathogenic bacteria *Lactococcus lactis*. This technique offers a unique and appealing method of assessing the simple, safe, economical, and ecologically friendly pilot manufacture of pharmaceutical items. The concept describes a workable way to effectively deliver a wider variety of protein antigens. To learn more about the immunogenicity and protective effectiveness of the L*. lactis*/pNZ8121-Multi epitope against virulent *salmonella* infection, we have initiated further study in our labs.

## Consent for publication

Not applicable.

## Funding

Not applicable.

## Supplementary material

Additional information related to this subject is available in the online version.

## CRediT authorship contribution statement

**Reyhaneh Sadat Moosavi-Kohnehsari:** Conceptualization, Writing – original draft, Writing – review & editing. **Mahnaz Jafari-Sohi:** Data curation, Writing – review & editing. **Tohid Piri-Gharaghie:** Conceptualization, Funding acquisition, Writing – review & editing. **Shakiba Tolou-Shikhzadeh-Yazdi:** Data curation, Software, Writing – review & editing. **Mona Aghassizadeh-Sherbaf:** Data curation, Writing – review & editing. **Romina Hosseinzadeh:** Data curation, Writing – review & editing.

## Declaration of competing interest

The authors declare that they have no conflict of interest.

## Appendix Supplementary materials

Image, application 1
